# Incorporating High-Dimensional Exposure Modelling into Studies of Air Pollution and Health

**DOI:** 10.1007/s12561-016-9150-3

**Published:** 2016-06-13

**Authors:** Yi Liu, Gavin Shaddick, James V. Zidek

**Affiliations:** 1 Department of Mathematical Sciences, University of Bath, Bath, UK; 2 Department of Statistics, University of British Columbia, Vancouver, Canada

**Keywords:** Air pollution, Health risks, Bayesian modelling, Spatio–temporal models, INLA

## Abstract

Performing studies on the risks of environmental hazards on human health requires accurate estimates of exposures that might be experienced by the populations at risk. Often there will be missing data and in many epidemiological studies, the locations and times of exposure measurements and health data do not match. To a large extent this will be due to the health and exposure data having arisen from completely different data sources and not as the result of a carefully designed study, leading to problems of both ‘change of support’ and ‘misaligned data’. In such cases, a direct comparison of the exposure and health outcome is often not possible without an underlying model to align the two in the spatial and temporal domains. The Bayesian approach provides the natural framework for such models; however, the large amounts of data that can arise from environmental networks means that inference using Markov Chain Monte Carlo might not be computationally feasible in this setting. Here we adapt the integrated nested Laplace approximation to implement spatio–temporal exposure models. We also propose methods for the integration of large-scale exposure models and health analyses. It is important that any model structure allows the correct propagation of uncertainty from the predictions of the exposure model through to the estimates of risk and associated confidence intervals. The methods are demonstrated using a case study of the levels of black smoke in the UK, measured over several decades, and respiratory mortality.

## Introduction

The background to this paper involves two major processes that are changing the world dramatically. The first is population growth: recent projections suggest that the World’s population would move from today’s 7 billions to 11 billions by the year 2100 contrary to earlier estimates that had seen it declining after year 2050 [25]. Since a large fraction of environmental hazards are anthropogenic in origin, this steep rise in population will have a serious effect on the environment. The second process, related to the first at least in part, is climate change. This is expected to generate more extreme weather events including heat waves [56]. Together these processes will have serious implications for human health and welfare [33].

Population growth and climate change are leading to an increased focus on policy making including mitigation strategies, management, regulation and control. This leads to an increased need for monitoring of environmental hazards in order to inform policy, together with accurate calculations of their potential effects. The amount of data that are available from environmental monitoring networks is growing all the time and is characterised by very high-dimensional records of measurements made over a large number of time points. As such there is a need for statistical tools that can encompass the information that is contained within these rich datasets. In the case study presented in this paper for example, data are available from several thousands monitoring sites. This leads to the central aim of this paper: an approach to modelling the effects of environmental exposures on health that can contend with these high-dimensional data records. Here we focus on the case of air pollution on health, although the methodology is general and could be applied to other environmental hazards.

Concerns about the potential adverse effects of air pollution on human health have been the subject of a great deal of research. Epidemiological studies have consistently reported associations between a variety of pollutants and both mortality and morbidity, including particulate matter [31], sulphur dioxide [51], nitrogen dioxide [72], carbon monoxide [12] and ozone [58]. Associations have also been shown within different sub-groups of the population, such as the elderly [16] and children [34] for a range of health outcomes, such as asthma [65] and respiratory and circulatory illnesses [28]. Determining whether safe levels exist is important for regulatory purposes and air pollution legislation such as the Clean Air Acts in the UK and US, which set safe levels for a number of common pollutants, and the WHO air quality guidelines [64], which offer global guidance on reducing the health impacts of air pollution.

Much of this evidence comes from studies of acute health effects, where short-term changes in exposure to air pollution are associated with increased mortality in subsequent days. A wide range of pollutants have been implicated in these effects, but particular attention has focused on particulate matter, measured in various ways (e.g. as PM$_{10}$, PM$_{2.5}$, total suspended particles and black smoke) and oxides of sulphur (SO$_x$).

Rather less attention has been given to investigating chronic effects of air pollution, i.e. the association between health outcomes and long-term exposures to air pollution, possibly over several years, due to the lack of availability of suitable data and issues related to confounding. However, it is uncertain to what extent, if at all, findings from studies of short-term effects can be extrapolated to longer term (chronic) effects. Whilst some or all of the effects of acute exposures may be attributed to ‘mortality displacement’, where health events which were likely to occur within a short time brought forward, chronic exposures may be implicated in fundamental disease causation, e.g. by sensitising people in early life to respiratory allergens or by provoking cell mutation.

The vast majority of the studies examining the associations between air pollution and health use data from monitoring networks as a proxy for the exposure to air pollution experienced by the populations in question. Information on ambient concentrations therefore often comes from a set of monitoring sites measuring pollutants over an extended period of time.

In order to perform health analysis using such data, there will be a need for accurate estimates of air pollution during periods and in locations when there are missing data. Missing data may be either by design, where a monitor is not located or in operation, or due to shorter periods where measurements are not available from a monitor. Commonly, simple methods for handling missing values are used including simply discarding them from the analysis or replacing them by a specific single value, for example, the overall mean. By discarding missing values, we may lose useful information and may in fact introduce bias. By replacing missing values by a single value, for example the posterior mean from an exposure model, important features of the data and the intrinsic variability in using a summary value may be ignored to the detriment of the quality of the estimates of adverse health outcomes.

In many epidemiological studies, the locations and times of exposure measurements and the health assessments do not match, in part because the health and exposure data will derive from completely different data sources and not as the result of a carefully designed study. This is known as *spatial misalignment* and an example can be seen in Lopiano et al. [38] who consider the case where data may be collected at different points or the same type of data at different frequencies leading to the need to synthesise them [69]. In addition, both continuous and discrete domains may have to be considered. For example, an environmental hazard, such as air pollution, might be thought of as a continuous process over space and time but health outcomes, i.e. counts, may only be available in aggregated form for administrative districts. This is termed the ‘change of support problem’ by Gelfand et. al. [24] and a direct comparison of the exposure and health outcome requires an underlying model to align the two in the spatial and temporal domains [26, 43].

A few studies have used spatial–temporal modelling within such health studies, largely due to the health data being available at a lower geographical temporal resolution than the exposure data [23, 32, 67, 71]. These studies are ecological in nature as the health data are likely to be available in aggregate form which means the exposures will likely need to be spatially or temporally aggregated. In such cases, there is potential for ecological bias where associations observed at the area level do not hold for the individuals within areas. Ecological bias can manifest itself in a variety of ways and here bias in the resulting health risks may occur due to the aggregation of a non-linear model [59–61].

The amount of monitored air pollution data that are routinely available is increasing dramatically; in London for example there are now more than 80 monitoring sites measuring particulate matter on an hourly, and sub-hourly basis compared with about 10 in the early nineties. Globally the WHO database on air pollution currently comprises ground-level measurements from 1600 cities. Being able to utilise the increasing amounts of available data will lead to more accurate assessments of levels of pollution and more realistic models through increased ability to investigate spatio–temporal dependencies.

A hierarchical modelling approach provides a natural way of modelling data with complex forms of dependence and the models presented here for that purpose are naturally set within a Bayesian framework. Modelling the entire spatio–temporal structure of an environmental field has often been impractical in the past due to the availability, or lack thereof, of data in the quantities required to produce reliable estimates. Where such data are available, its high dimensionality has meant that the computation required may be prohibitive. There is therefore a need for efficient methods of estimation, particularly in reference to the computational issues that are likely to arise when attempting to fit the models using Markov Chain Monte Carlo (MCMC) sampling. This has led to the development of alternative methods based on approximations within the Bayesian inferential framework and here we specifically consider those based on integrated nested Laplace approximations (INLA)[46].

The remainder of this paper is organised as follows. Section 2 contains details of health–exposure models for estimating the risk associated with cumulative exposure to air pollution. Section 3 presents a general approach to exposure modelling within a hierarchical framework, assuming an underlying process model of which measurements can be made (with error). This underlying process model may have structure in both time and space. In any Bayesian analysis, there will be another level; that of priors for the parameters. Details of these and possible methods for inference, notably the use of INLA, are described at the end of this section. In Sect. 4, we describe an approach for linking the spatio–temporal exposure models with health effects models. Here, predictions from the exposure models are used for locations in time and space where exposures are not available but where health outcomes are available. Section 5 contains a case study that demonstrates the use of our proposed approach to exposure modelling and its integration with health analyses. Long-term concentrations of black smoke, a measure of particulate matter, measured over several decades in the UK are related to respiratory mortality leading to the estimation of the adverse effects on health. Section 6 provides a concluding discussion and some suggestions for future research.

## Preliminaries

In this paper, we develop an approach for estimating the adverse health effects of environmental hazards. This approach incorporates health data, available in aggregate form, and exposure data measured at point locations over space and time. We particularly consider cases where spatio–temporal data records are of such high dimension that conventional computational approaches fail. Before embarking on the description of the health and exposure models, we now describe the general framework of hierarchical Bayesian models and the notation that will be used throughout the paper.

Hierarchical Bayesian models are an extremely useful and flexible framework to model complex relationships and dependencies in data. There are three parts to the hierarchy:

- The observation, or measurement, level; $Y | Z, X, \theta _1$. The data are assumed to arise from an underlying process which is unobservable but from which measurements can be taken, with error, at locations in space and time. Measurements may also be available for covariates, *X*.
- The underlying process level; $Z | \theta _2$. This drives the measurements seen at the observation level. It may be, for example, a spatio–temporal process representing an environmental hazard.
- The parameter level; $\theta = (\theta _1, \theta _2)$. This contains models for all of the parameters in the observation and process level and may control things such as the variability and strength of any spatio–temporal relationships.

Here the notation *Y* | *X* means that the distribution of *Y* is conditional on *X*. The underlying spatio–temporal process, *Z* may be viewed as lying in continuous domains of time and space, $\mathcal{T}\subset \mathcal{R}$ and $\mathcal{S}\subset \mathcal{R}^d$ respectively, where $\mathcal{R}^d$ denotes a *d*-dimensional Euclidean space. However, even when *Z* is continuously monitored over time, monitors may only report results at discrete times, i.e. $\mathcal{T} = \{0,1,\dots ,N_T\}$ for some $N_T$. The same may be true over space, where the locations where air quality monitors can actually be placed may be restricted to a relatively small number of locations, for example, on public land, leading to a discrete $\mathcal{S}$ in practice.

The approach developed in this paper involves models for both health counts as well as exposures and each of these can be framed in the context of a hierarchical model. To avoid ambiguity between the two, we use $Y^{(1)}, X^{(1)} , Z^{(1)}, \theta ^{(1)}$ for the health models and $Y^{(2)}, X^{(2)} , Z^{(2)}, \theta ^{(2)}$ for the exposure models. It is noted that although the health counts, $Y^{(1)}$, can be considered to be measurements from an underlying true level with differences occurring, for example, due to misclassification or data anomalies, here we consider them to be an accurate reflection of the truth, i.e. $Y^{(1)} = Z^{(1)}$.

## Health Effects Models

In order to assess the effect of air pollution on health, models are required that relate risk to the exposure, both in terms of the degree of exposure and the time over which the exposure occurred. In cohort studies of individuals, such models need to account for the duration of exposure, time since first exposure, time since exposure ceased and the age at first exposure [5, 62]. For the development of carcinogenesis, complex multistage models have been developed that use well-defined dose–response relationships [13]. However, when using aggregated daily mortality counts for a specific period, e.g. day or health period, and specified area, detailed exposure histories and other information are generally not available.

Considering a generic area for ease of illustration, let $Y^{(1)}_t$ be the health outcome at time *t*, e.g. the number of respiratory deaths on a single day or other period of time, and the true exposure history ${Z_u, 0 \le u \le t}$, then the outcome is modelled as a function of the exposure history.

1 $$\begin{aligned} E(Y^{(1)}_t) = f(Z^{(2)}_u; 0 \le u \le t). \end{aligned}$$

As true lifetime personal exposure to air pollutants is unmeasurable, it being dependent on ambient levels and integrated time activity, the term ‘exposure’ here relates to cumulative ambient outdoor concentrations of air pollutants, measured at the aggregate area level. The summaries of the exposure history are therefore constructed based on available data, $Y^{(2)}_t$.

If it is assumed that $Z_u$ is piecewise continuous, then the cumulative postnatal exposure up to and including time *t* is

2 $$\begin{aligned} \int ^{u=t}_0 Z^{(2)}_u du. \end{aligned}$$

Rather than just considering the effect of the total exposure over a period of time, the contributions from intervals within the period may be of interest, in which case Eq. (2) can be expressed in the form of weighted integrals [1, 6].

3 $$\begin{aligned} C_t = \int ^{u=t}_{o} W_{t-u} Z^{(2)}_u du, \end{aligned}$$

where the weights, $W_{t-u}$, determine the aspect of the postnatal exposure being summarised. For example if the weights are of the form $W_u =$ min(1, *u* / *b*) or 0 according as $u>0$ or $u\le 0$, then the exposures are phased in linearly over a period of length *b* until reaching their maximum. This can allow for delayed as well as cumulative effects depending on the form of the weights. In individual studies that focus on postnatal exposure, the form of the cumulative exposure can be explicitly modelled, for example in the case of exposure to asbestos fibres, where the rate of elimination of the fibres from the lungs, $\lambda$, may be incorporated and the model takes the form $W_u = \{1-\exp (-\lambda u)\}/\lambda$ [3].

The exposure of interest is likely to be over a specified period of time and if the weights are of the form

4 $$\begin{aligned} W_u = \left\{ \begin{array}{lll} 1/(b-a) &{} \text{ for }&{} 0< a \le u < b \\ 0 &{} \text{ otherwise } \end{array} \right. \end{aligned}$$

then the summary will represent the average for the period $(t-b,t-a], 0 \le a<b \le t$. For example, when studying the short-term effects of air pollution, with daily measurements of health and air pollution, if $a=0$ and $b=2$, then $W_{t-u}$ would represent a three day mean.

When dealing with health counts, and exposure measurements, made at discrete times, the integral in Eq. (3) can be approximated by a summation over a suitable discretisation

5 $$\begin{aligned} C_t = \sum ^{t}_{k=0} W_{t-k} Z^{(2)}_k. \end{aligned}$$

If the probability of disease given cumulative exposure is assumed to be proportional to $\exp ( \gamma C_t )$, i.e. a log-linear model in cumulative exposure, then a Poisson model can be used to estimate the weights, $W_{t-k}$ in Eq. (5). Assuming that $Y_t \sim P(E_t \mu _t)$ where $E_t$ represents the expected number of cases [7] then

6 $$\begin{aligned} \log \mu _t= & {} \beta _0+\gamma \sum ^t_{k=0} W_{t-k} Z^{(2)}_k + \sum ^{P}_{p=1} \beta _{p}X^{(1)}_{pt} \nonumber \\= & {} \beta _0+\sum ^t_{k=0}\beta _{t-k}Z^{(2)}_{t-k} + \sum ^{P}_{p=1} \beta _{p}X^{(1)}_{pt} \end{aligned}$$

where $X^{(1)}_p,~ p= 1, \dots P$ are area-level covariates. Hence the parameters, $\beta _{t-k}$ represent the effect of exposure *k* time periods ago. Comparing with Eqs. (3) and (5) shows that $\beta _{t-k} = \gamma W_{t-k}$. The expected number of deaths will be $E = \sum _{k=1}^K N_k r^\prime _k$, where $r^\prime _k$ are the age–gender-specific mortality rates for the reference population (usually a country or other large area) and $N_k,~ k=1,\ldots ,K$ are the populations in the area of study, in each age–gender group *k*. It should be noted that these are not the expected number of cases in the sense of statistical expectation, but are what would be expected based on applying national rates of disease to the population structure of the areas being studied.

It is possible to specify the shape of the distributions of the weights, $W_{t-k}$. For example, Schwartz et. al. [52] describe the use of a distributed lag model (DLM) within aggregate level studies examining the short-term effects of air pollution on health where the weights fit a polynomial function [29]. This requires assumptions to be made on the maximum lags that are likely to have an effect and the smoothness of the patterns over lags, which is determined by the polynomial used, but has the advantage of increasing the stability of the individual estimates when there is high collinearity between the explanatory variables [66]. The required assumptions have been formulated in terms of priors when implementing DLMs within a Bayesian setting [63].

There is a strong possibility of over dispersion in the Poisson models, where the variance is greater than the mean, arising from the presence of unmeasured confounders. These may be operating at the individual level, e.g. smoking, or at the area level, e.g. residual socio-economic confounding. Over dispersion may also arise because of data anomalies, i.e. errors in the numerators and/or denominators, e.g. due to migration which may make it unreasonable to assume that $Y^{(1)} = Z^{(1)}$. Making no allowance for the extra-Poisson variability that may be present will lead to confidence intervals for the estimates of risk being too narrow and changes in deviances, used to compare models, being too small. An attempt to correct these effects can be made using quasi-likelihood [40] or, in a Bayesian setting, by incorporating random effects within the model (see [19] for an example of this).

## Exposure Modelling

As discussed in Sect. 1, there will often be missing values in the available exposure data. These will arise both from short periods in which monitors were not reporting information and from locations and times for which there were no monitoring sites. One approach is to represent the ambient pollution surface with a spatial or spatio–temporal model, and then to estimate the quantities of interest such as estimated exposures when and where measurements were not taken using prediction methods. As described in Sect. 2, the spatio–temporal random field, $Z_{st}, s \in \mathcal{S}, t \in \mathcal{T}$, is a stochastic process over a region and time period. This underlying process is not directly measurable, but realisations of it can be obtained by taking measurements, possibly with error, at a set of known locations in space $S =\{s_1,\ldots ,s_{N_S}\} \in \mathcal{S}$ and time $T =\{t_1,\ldots ,t_{N_T}\} \in \mathcal{T}$. In a purely spatial analysis, repeated observations at a specific location over time are treated as independent realisations of the underlying process.

The observed data, $Y^{(2)}_{st}, s=1,\ldots ,N_S, t = 1,\ldots ,N_T$, at the first level of the model are considered conditionally independent given a realisation of the underlying process, $Z^{(2)}_{st}$. The second level describes the true underlying process as a combination of a trend (mean), $\mu _{st}$, and a random process, $\omega _{st}$, which has spatial–temporal structure in its covariance. In a Bayesian analysis, the third level of the model assigns prior distributions to the hyperparameters from the previous levels. Thus in summary, we have

7 $$\begin{aligned} Y^{(2)}_{st}= & {} Z^{(2)}_{st} + \epsilon _{st} \nonumber \\ Z^{(2)}_{st}= & {} \mu _{st} + \omega _{st} \end{aligned}$$

where the $\{ \epsilon _{st}\}$ is a set of independent random, or measurement, error terms, $\mu _{st}$ is a space-time mean field (trend) and $\omega _{st}$ is a spatial–temporal process.

The second line in Eq. (7) comprises a mean function together with a zero-mean spatial–temporal process. Previous studies have modelled the mean function with a trend surface model [67], cyclical variation [57], a temporal only trend [53, 68] and the Kriged-kalman model [49]. The spatial–temporal process can be considered to be the combination of three components; space, time and space–time interaction. These three components may be combined in either additive or multiplicative form [49, 67]. For the former, we have

8 $$\begin{aligned} \omega _{st}= m_s + \gamma _t + \kappa _{st}. \end{aligned}$$

This form has been used by a number of authors to model ambient air pollution, including for example [48, 49, 68] and [47] who modelled PM$_{10}$ in Vancouver, Canada and PM${_{2.5}}$ in Ohio state, New York City and a collection of midwestern states in the US, respectively. In a *separable* model, the spatial and temporal components are considered entirely separately with no interaction between them, i.e. $\kappa _{st}=0$.

It is commonly assumed that the spatial effects, $m_s$, represent a stationary spatial process with the relationship between correlation and distance between the sites being represented by a function from the Matern family of covariance functions, which takes the following form:

9 $$\begin{aligned} \frac{\sigma ^2}{2^{\nu -1}\Gamma (\nu )}(2\sqrt{\nu } t\phi )^{\nu }K_{\nu }\sqrt{\nu } t\phi \end{aligned}$$

where $K_{\nu }(\theta \parallel h \parallel )$ is a modified Bessel function of the second kind, $\sigma ^2$ is the overall variance and $(\nu , \phi )$ are parameters that control the smoothness and strength of the distance–correlation relationship, respectively.

### Prediction at Unsampled Locations

The posterior predictive distributions for the underlying level at a point $s_0$ not included in the sampled locations are

10 $$\begin{aligned} p(Z^{(2)}_0 | Y^{(2)}) \propto p(Z^{(2)}_0, Y^{(2)})= & {} \int ... \int p(Y^{(2)},Z^{(2)} , Z^{(2)}_0 )dZ^{(2)} \nonumber \\= & {} \int ... \int \left\{ \prod ^{N_S}_{s=1}p(Y^{(2)}_{s} |Z^{(2)}_s)\right\} \times \dots \end{aligned}$$

11 $$\begin{aligned} \dots\times & {} p(Z^{(2)}_0|Z^{(2)})p(Z^{(2)})dZ^{(2)}. \end{aligned}$$

This form can be further expanded to incorporate the conditioning on the parameters within the model, i.e. $p(Z^{(2)}|\psi , \nu , \phi )$, where $\psi$ are the coefficients in the mean term and $(\nu , \phi )$ those in the variogram/covariance function. In this way the uncertainty in the estimation of the parameters of the spatio–temporal model can be ‘fed’ through to the predictions.

#### Inference

For Bayesian analyses, the posterior distributions will often involve high-dimensional integration and may be analytically intractable. However, samples from these distributions may theoretically be generated in a straightforward fashion using MCMC sampling [55]. The main constraint for this approach, particularly when using large spatial datasets, is its demanding computational requirements. This can be both because of the need to manipulate large matrices within each simulation of the MCMC and also due to the lack of convergence of parameter estimates in complex models [21].

The increasing size and complexity of experiments and the databases they generate have outpaced the speed of readily available computational hardware. This has forced the development of practical alternatives to MCMC algorithms. One approach is to marginalise out the spatial effects details of which can be found in Finley et. al. [21]. The basic idea is to use a simpler model with the spatial effects component marginalised out. The covariance is then a combination of the random and (spatial) structured effects, $\Xi =\sigma ^2_m\Sigma _{m(\phi )}+I\sigma ^2_u$, where $\sigma ^2_m$ is the overall spatial variance, $\Sigma _{m(\phi )}$ is the spatial covariance which will be dependent on the parameter $\phi$ which determines the relationship between correlation and distance, *I* is the identity matrix and $\sigma ^2_u$ is the random variation. The idea is that this matrix is more stable than $\sigma ^2_m\Sigma _{m(\phi )}$ and it is expected that the posterior distribution of $\phi$ will converge. This means that most of the full conditionals of the parameters cannot be found in closed form and here a Metropolis–Hasting step is used for the variables $(\sigma ^2_u,\sigma ^2_m,\phi )$, where $\phi$ represents the strength of the decay in the correlation–distance relationship. The spatial effects are marginalised out, which reduces the parameter space and thus lessens the computational burden. However, this means that estimates of the spatial effects, which are required for prediction, are not available as they cannot be sampled. In the Gaussian case, they can be reconstructed in a posterior predictive fashion [2].

Here we concentrate on recently developed techniques, which perform approximate Bayesian inference based on integrated nested Laplace approximations (INLA) and thus do not require full MCMC sampling to be performed [46]. INLA has been developed as a computationally attractive, practical alternative to MCMC.

INLA uses a Laplace approximation to the posterior distribution of the parameters, $\theta$, given measurements of the response, *Y*. For clarity of exposition, we drop the $Y^{()}, Z^{()}, \theta ^{()}$ notation in what follows. When the process model is Gaussian, we have a latent Gaussian model:

- Observation model; $$y_{st} | z_{st} \sim \pi (y_{st} | z_{st}, \theta _1)$$.
- Process model; $$Z | \theta _2 \sim N(\mu , \Sigma _{\theta _2})$$.
- Parameter model; $$\theta = (\theta _1, \theta _2) \sim \pi (\theta )$$.

Therefore $\pi (Z, \theta | y) \propto \pi (\theta ) \pi (Z |\theta )\prod ^{N_T \times N_S}_j \pi (y_{j} | z_{j}, \theta )$. In the setting, considered here, the response consists of measurements of air pollution which are assumed to depend stochastically on a latent process, *Z*, which is indexed by spatial–temporal locations, *st*. If the latent process can be expressed as a Gaussian Markov Random Field (GMRF) then, with Gaussian observations, the resulting joint distribution will be a GMRF. For a GMRF, the precision matrix, $Q_\theta = \Sigma ^{-1}_{\theta }$ will be sparse, allowing efficient computation.

The aim is to obtain posterior marginal quantities such as $\pi (\theta _i | \mathbf y )$ and $\pi (z_{st} | \mathbf y )$ where for example $\pi (\theta _i | \mathbf y ) = \int \pi (\theta | \mathbf y ) d\theta _{-i}$ and $\pi (z_{st} | \mathbf y ) = \int \pi (\theta | \mathbf y ) \pi (z_{st} | \theta , \mathbf y ) d\theta$. In order to achieve this, approximations need to be built; $\tilde{\pi }(\theta | \mathbf y )$ and $\tilde{\pi }(z_{st} | \theta , \mathbf y )$.

The Laplace approximation to the posterior $\tilde{\pi }(\mathbf {\theta } | \mathbf y )$ is given by

$$\begin{aligned} \tilde{\pi }(\mathbf {\theta } | \mathbf y ) =\propto \left. \frac{\pi (\mathbf z , \mathbf {\theta } , \mathbf y ) }{\tilde{\pi }_G(\mathbf z | \mathbf {\theta } , \mathbf y ) } \right| _{ z= z^*(\mathbf {\theta } )} \end{aligned}$$

where $\tilde{\pi }_G$ is a Gaussian approximation at the mode $z^*(\mathbf {\theta } )$ of the conditional distribution of $\mathbf z$ given $\mathbf {\theta }$. Given such an approximation, numerical integration can be used to evaluate the required integral. The same procedure can be used to approximate the posterior distribution of $\pi (z_{st} | \mathbf y )$.

The accuracy to which INLA can compute approximations to the posterior marginal distributions is well documented, see for example [46] and [50]. Whilst INLA is usually very accurate, [20] have shown some cases with binomial and Poisson data where a correction may be required, although it is noted that these are very extreme cases. A number of authors have recently reported favourable comparisons with results obtained using MCMC including [42], who fit a bivariate meta-analysis of diagnostic test accuracy studies and [22], who perform comparison for a variety of examples. When comparing the results from OpenBUGS and R-INLA in a disease mapping setting, [9] found some differences in the estimates of the random effects and their precisions when using R-INLA with the default priors, but found that exact replicates could be found by specifying alternatives.

In a spatial setting the INLA approach provides a natural approach to modelling areal level data. Applying the approach to point level data of the type that will arise from air pollution monitoring sites can be performed by using a link between Gaussian Fields (GFs) with Matern covariance functions and GMRFs through use of stochastic partial differential equations (SPDE) [35].

Lindgren et. al. [35] show that a field with a Matern covariance structure can be expressed as the solution of an SPDE. If a GF, *Z*, has a Matern spatial covariance as given by (9) then it is the solution of the SPDE

12 $$\begin{aligned} (\kappa ^2 - \Delta )^{\alpha / 2} z_{S} = \mathcal{W}_{S}, S \in \mathcal{S}, \alpha = \nu + d/2, \kappa> 0, \nu > 0, \end{aligned}$$

where $(\kappa ^2 - \Delta )^{\alpha / 2}$ is a pseudo-difference operator, $\Delta$ is the Laplacian and $\mathcal{W}$ is spatial white noise with unit variance.

This SPDE in turn can be approximated using a finite element method, using triangulation over the spatial domain of interest. An induced GMRF representation of the original GF can then be found with the precision matrix being approximated by a sparse precision matrix, *Q* . This represents the information within the covariance matrix of the original GF, $\Sigma$ and its sparsity allows computational efficiency. The GMRF is used by INLA for performing computations that would be computationally prohibitive using the GF directly.

## Linking Exposure and Health Models

Pollution data are generally obtained from $N_S$ fixed site monitors located within $\mathcal {S}$, each of which will measure ambient pollution concentrations continuously throughout the year. The set of pollution monitoring sites are collectively denoted by $S = s_1, \dots , s_{N_S}$, where $s_l = ( a _{ l } , b _{ l }) \in \mathcal {R}^2$. However, health data are commonly available only at aggregated level for administrative areas, $A_i, i=1,\ldots ,N_A$ and therefore a suitable summary of the concentrations in an area for a particular time period is required. The true mean exposure for time *t* in a health area, $A_i$ is given by

13 $$\begin{aligned} \overline{z}_{it}\ =\ \int _{s \in \mathcal {A}_i} N_s z_{st} ds, \end{aligned}$$

where $z_{t\mathbf {s}}$ is the ambient pollution concentration at all possible locations *s* in $A_i$ at time *t* and $N_s$ is the population density such that $\int _{s \in \mathcal {A}_i} N_s ds = 1$. However, the information required to perform the integral will be unavailable. Therefore, there is a need to approximate this, with the simplest and most commonly used approach being to take the average of the observed measurements from actual monitoring sites located within the health area,

14 $$\begin{aligned} \bar{y}_{it} = \frac{1}{ N_{A_i} }\ \sum _{s \in A_i} y_{st}, \end{aligned}$$

where $N_{A_i}$ is the number of monitoring sites located within area $A_i$. Here missing values are typically ignored, something that can lead to bias if there are strong temporal trends in the data. An example of this can be seen in the case study presented in Sect. 6.

Alternatively, an exposure model can be used to provide the required information including using predictions in place of any missing values. Any approach for using such predictions in the health model must acknowledge the uncertainty in the predictions and allow for it to be incorporated in final measures of uncertainty, and confidence intervals, associated with those measures of risk.

In a fully Bayesian analysis, estimation for both the health and exposure models, including prediction at locations where data are not available, would be performed simultaneously. The uncertainty in estimating the coefficients of the exposure model is therefore acknowledged and ‘fed through’ the model to the predictions and thus to the estimation of the coefficients in the health model.

There are likely to be computational considerations associated with jointly fitting the health and exposure models, especially if the latter uses large amounts of data over space and time. When the exposure model is complicated or when one is interested in running multiple candidate epidemiological models with different sets of covariates either for a single outcome or multiple outcomes, a single model is not going to provide an efficient method of investigation.

Often the exposure models are fit separately from the health model, removing the dependence between *Y* and *Z*, in order to ease the computational burden in running a combined model, an approach that has also been adopted in Carlin et. al. [8] and Zhu et. al. [67]. This *two-stage approach* has the advantage that the exposure model, which is likely to be the most computationally demanding, does not have to be refit when running multiple health effect analyses. Two- stage approaches separate the exposure and health components, whilst still allowing uncertainty from the exposure modelling to be incorporated into the health model [11, 32, 43].

There are other reasons why fitting a joint model may be unappealing; it is not intended that the health counts should inform the estimation of the exposures which should be based on data from the monitored concentrations. It is possible to ‘cut’ feedback between the stages within MCMC, for example in WinBUGS [39]. This may be achieved by simplifying the full conditional distributions by removing the dependence on the health data for those parameters associated with the exposure model; however, the result is that the posteriors may not be proper probability distributions [44].

### Multiple Imputation

One approach to performing a two-stage analysis is to use multiple imputation [37]. This allows the uncertainty in predictions to be represented by using set of plausible values for the exposures, which comprise samples from the posterior distributions of the predictions at the required locations in space and time. Taking *M* multiple (joint) samples from the posteriors results in *M* multiple datasets which are repeatedly used in the health model.

This requires the ability to draw joint samples from the posterior distributions of the predictions from the exposure model. This is possible in the R-INLA package using the function inla.posterior.sample. In computing the approximation to the required distributions, $\tilde{\pi }(\theta |\mathbf {y})$ $\tilde{\pi }(z_{st} |\theta , \mathbf {y})$, R-INLA uses numerical integration based on interpolation between a number of chosen ‘integration points’ [46]. Taking $\tilde{\pi }(z_{st} |\theta , \mathbf {y})$ as an example, the integration points are selected from a set of candidate points on a grid. After exploring $\log (\tilde{\pi }(z_{st} |\theta , \mathbf {y}))$ to find the mode, a point is selected if the difference between $\log (\tilde{\pi }(z_{st} |\theta , \mathbf {y}))$ evaluated at that point and the value evaluated at the mode is greater than a prespecified constant. Apart from the integration based on this procedure for finding approximations to the marginal distributions as described in Sect. 4.1.1, the information stored about the distribution at these integration points can be kept. This allows the function inla.posterior.sample to be used after the main INLA run. Joint samples from the posteriors can be obtained by sampling from Gaussian approximations at the integration points for all of the parameters, including predictions from the exposure model. A combined analysis of these datasets is then performed. This results in valid statistical inferences that properly reflect the uncertainty due to missing values.

Repeatedly running the health model results in an estimate of the log relative risk, $\beta _{1}$, and associated standard error for each dataset. These are then combined to give an overall estimate of relative risk together with a combined standard error that can be used to calculate confidence intervals [45]. Assume $\beta _{1d}$ is the estimate obtained from dataset *d* (d =1,2,...,n) and $\sigma _{\beta d}$ is the standard error associated with $\beta _{1d}$. The overall estimate is the average of the individual estimates,

15 $$\begin{aligned} \bar{\beta }_1\ =\ \frac{1}{n} \sum _{1}^{n} \beta _{1d} \end{aligned}$$

The overall estimate of the standard error will be a function of a combination of within-imputation variance and between-imputation variance. The first of these is given as

$$\begin{aligned} \sigma _{w\beta }^2\ =\ \frac{1}{D} \sum _{1}^{D} \sigma _{\beta d}^2 \end{aligned}$$

and the between-imputation variance by

$$\begin{aligned} \sigma _{b\beta }^2 =\ \frac{1}{n-1} \sum _{1}^{n} (\beta _{1d} - \bar{\beta }_{1d})^{2}. \end{aligned}$$

The total variance is therefore

$$\begin{aligned} \tau ^2\ = \sigma _{b\beta }^2\ + \ ( 1 + \frac{1}{D} ) \sigma _{w\beta }^2. \end{aligned}$$

Confidence intervals are obtained using quantiles of the t-distribution with degrees of freedom

$$\begin{aligned} df\ =\ ( D - 1 )\ \Bigg ( 1 + \frac{D \sigma _{b\beta }^2}{(D + 1) \sigma _{w\beta }^2} \Bigg )^{2}. \end{aligned}$$

## Case Study

**Fig. 1:**
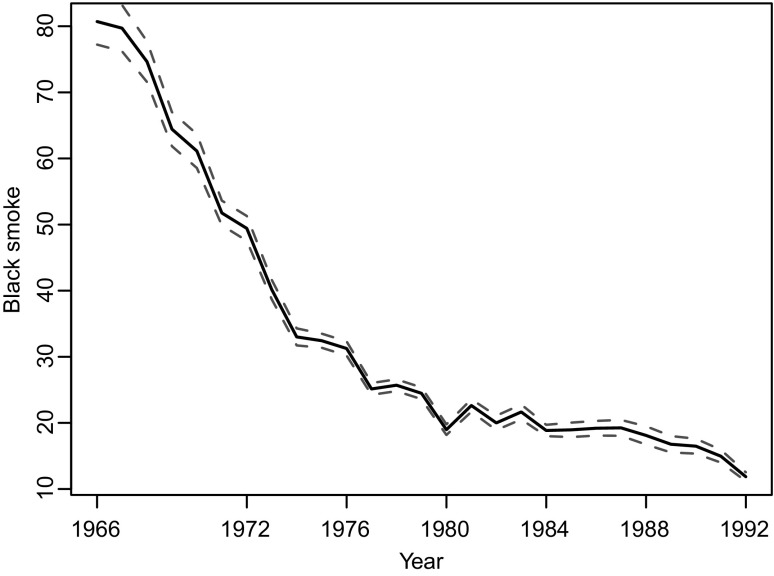
Average concentrations of Black Smoke ($\mu$gm$^{-3}$) (*black line*) from 1966 to 1992 with associated $95\,\%$ confidence intervals (*red dotted lines*) (Color figure online)

The UK black smoke and sulphur dioxide network measured black smoke (BS) and sulphur dioxide (SO$_2$) from the early 1960s until 2006. During that time, at its peak it comprised of over 1200 sites (in the early 1970s). As levels of BS and SO$_2$ declined from the very high levels in the 1960s, the network dramatically reduced in size and by 2005, shortly before it ceased operation, it contained 65 sites. Over this time, there was a marked decline in the average concentrations of BS which can be seen in Figure 1. For further details of the long-term changes in levels of BS and changes in the network see Shaddick and Zidek [54].

**Fig. 2:**
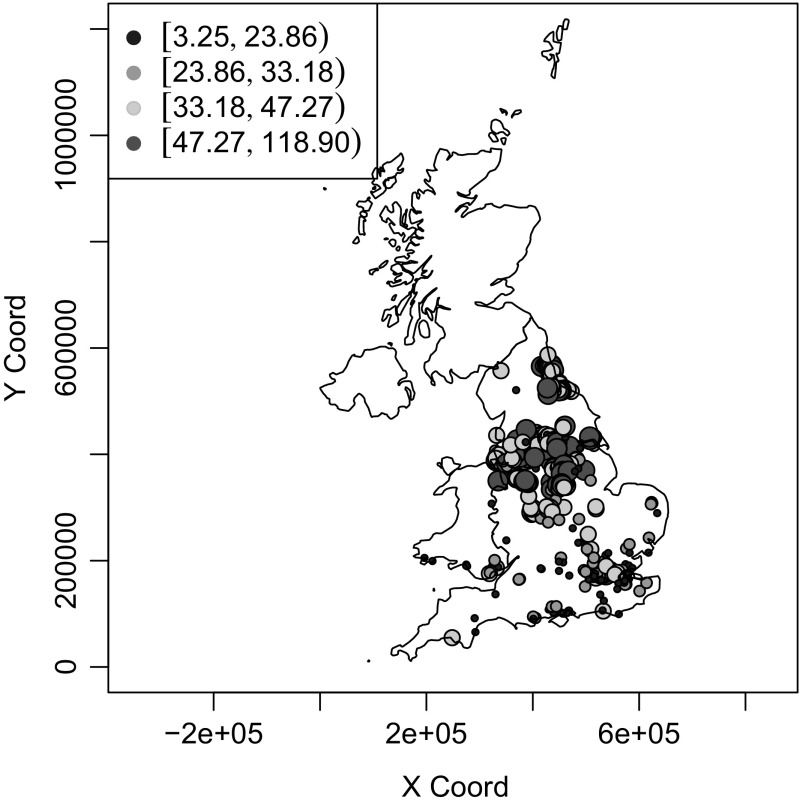
Average concentrations of black smoke ($\mu$gm$^{-3}$) measured at monitoring sites within the UK, 1996–1992

Data were obtained for a total of 3016 sites throughout the operation of the network, of which 2137 sites were designated as being located in residential areas. The locations of the monitoring sites were linked using GIS, as described in [19], to electoral wards which is the resolution of the health data. The locations of the wards, together with an indication of the average concentrations of BS over the study period are shown in Fig. 2.

This allows analyses of the association between health and air pollution to be performed; however, there will be areas in which health data are available but monitoring information was not available. The health data consist of mortality counts for the period 1993–1996 for respiratory diseases in the over 65 s. These data were extracted for all ages by ward from national postcoded mortality data, by age and sex, for the period 1993–1996. Expected numbers, standardised by age and sex, were calculated for each ward using national mortality counts and population data from the 1991 census. Smoking is known to be a major risk factor for cardio-respiratory illness and it is known that smoking habits vary with social class [30] and may therefore correlate with pollution levels, and act as a potential confounder. In the absence of data on smoking levels, an area-level measure of socio-economic deprivation is used [10], which has previously demonstrated to be related to smoking rates [30].

The period of study is chosen to represent a time (for the health period) which follows an extended period during which there were great changes in the levels of BS. Studies of the chronic effects of pollution have largely considered concurrent exposures. Over recent decades, air pollution concentrations have generally fallen, in response to industrial and technological changes and more rigorous regulation. At the same time, the character of air pollution has changed markedly, as domestic and industrial coal-burning has declined and emissions from road traffic have increased. Health risks determined on the basis only of current or recent exposures may therefore be misleading, especially for older age groups who may in the past have been subject to very different exposure regimes. Here we use exposures over the previous 27 years.

### Statistical Modelling

#### Exposure Modelling

Let $Y^{(2)}_{st}$ be the concentration of black smoke measured at location, *s*, at time, *t*. Ott [41] has suggested that a log transformation is appropriate for modelling pollution concentrations, because in addition to the desirable properties of right-skew and non-negativity, there is justification in terms of the physical explanation of atmospheric chemistry. We adopt a similar model to that presented in Shaddick and Zidek [54] and model the change in levels of BS over time using a random effects model with the quadratic relationship between time and concentrations of BS.

16 $$\begin{aligned} Y^{(2)}_{st} =(\beta ^{(2)}_{0} + \beta ^{(2)}_{0s}) + (\beta ^{(2)}_{x} + \beta ^{(2)}_{xs}) t + (\beta ^{(2)}_{x^{2}} + \beta ^{(2)}_{x^{2}s}) t^{2} + \epsilon _{st}, \end{aligned}$$

where $s = 1, \ldots , N_S$ denotes the site and $t = 1, \ldots , N_T$ the year. Note that the superscript, $\beta ^{(2)}$ refers to the fact that these parameters are for the exposure model and distinguishes them from parameters in the health model shown in Sect. 6.1.2 that have $\beta ^{(1)}$. The model includes both linear and quadratic effects, $\beta ^{(2)}_{x}$ and $\beta ^{(2)}_{x^{2}}$ of time reflecting the shapes of decline in the decline in levels of black smoke observed in the data. The $\epsilon _{st}$ is a random error term, which is assumed to be Normally distributed, $\epsilon _{st} \sim N(0 , \sigma _{\epsilon }^{2})$. Site-specific random effects, $\beta ^{(2)}_{xs}$ and $\beta ^{(2)}_{x^{2}s}$ and $\beta ^{(2)}_{0s}$, are assigned to the slopes of the linear, quadratic and intercept components, respectively. Each of these set of random effects is constrained to sum to zero and centred on the corresponding fixed effects, $\beta ^{(2)}_{0}$, $\beta ^{(2)}_{x}$ and $\beta ^{(2)}_{x^{2}}$. After allowing for the effects of time, there is likely to be spatial structure in the residuals and therefore the random effects are multivariate normally distributed, $\beta ^{(2)}_{0s} \sim MVN(0 , \sigma ^2_{s_{\beta 0}}\Sigma _{0s}), \beta ^{(2)}_{xs} \sim MVN(0 , \sigma _{s_{\beta _x}}\Sigma _{\beta _x}),\beta ^{(2)}_{x^{2}s} \sim MVN(0 , \sigma ^2_{s_{\beta _x2}} \Sigma _{\beta _x2})$, with the structure of the covariances reflecting any spatial auto-correlation as in Eq. (9).

#### Health Modelling

Expanding Eq. (6), we model the number of counts in area *i* for time *t* (defined as 1993–1996 for this analysis rather than a single year) as Poisson, $Y^{(1)}_{it} \sim P(E_i \mu _{it})$, where $E_i$ represents the expected number of cases in area *i* for the period from which the health data arise. The log of the rate, $\mu _{it}$ is modelled as a function of the levels of air pollution over the previous 27 years with the area-level covariate, $X^{(1)}_2$ representing deprivation. In principle, this could be a time-varying covariate, but in this example it is measured at a single point, using information from the 1991 census. As in Eq. (6), we use equal weights for each year and use the average pollution over the chosen time period for each area:

17 $$\begin{aligned} \log \mu _{it} = \beta ^{(1)}_0 + \beta ^{(1)}_1 \tilde{Z}_{s_i}^{(2)} + \beta ^{(1)}_{2}X^{(1)}_{2i}, \end{aligned}$$

where $X^{(1)}_2$ is the area-level index of deprivation with associated coefficient $\beta ^{(1)}_2$ and $\beta ^{(1)}_{1}$ represents the effect of the previous 27 years of exposure which for area $A_i$ is represented by $\tilde{Z}_{i}^{(2)}$.

Estimates of the exposure for each area, $\tilde{Z}_{i}^{(2)}$ can be obtained in a number of ways and here we consider three methods:

*M1*: The average of the available data.
*M2*: The average of predictions from the spatio–temporal exposure model.
*M3*: A combination of available data and predictions from the exposure model, enabling missing data to be ‘filled in’.

In both M2 and M3, there will be 27 values used in calculating the average over the previous 27 years. However, in M1 the fact that data may be missing is ignored and takes no account of the fact that in some cases the average may be based on small numbers. When there are clear trends in the data, as there are here, the times at which data are available may strongly affect the resulting summary of exposure. For example, if levels are decreasing then missing data at the beginning of the period will result in an underestimate of the overall exposure as higher values will be excluded. Similarly, missing data in the later period when exposures are lower would result in an overestimate.

For methods M2 and M3, multiple imputation is performed by drawing samples from the posterior distributions of the predictions in order to acknowledge the uncertainty that is associated with predicting from the exposure model. One hundred sets of data were produced, comprised of either a combination of available data and predictions (M3) or just predictions (M2).

## Results

Table 1 shows the estimated relative risks per 10 ${{\upmu }}$gm$^{-3}$ of black smoke together with their corresponding $95\,\%$ confidence intervals obtained from applying the three approaches described in Sect. 6.1.2. For each approach, relative risks are estimated with and without adjustment for deprivation. Results for methods M2 and M3 are obtained from multiple imputation of 100 samples from the joint posterior distribution of the exposure predictions.

**Table 1:** Relative risks (RR) of respiratory mortality, with 95 % confidence intervals for an increase of 10 ppb of BS over the previous 27 years

Without deprivation		With deprivation	
Method 1: observed exposures only			
RR	95 % CI	RR	95 % CI
1.037	1.025–1.050	1.038	1.023–1.049
Method 2: predictions			
RR	95 % CI	RR	95 % CI
1.022	1.014–1.030	1.021	1.013–1.029
Method 3: observed data and predictions combined			
RR	95 % CI	RR	95 % CI
1.011	1.004–1.018	1.010	1.003–1.017

Exposure values are obtained using three methods: (1) using observed data; (2) using predictions from a spatio–temporal model; (3) using observed data combined with predictions to fill in missing values. Risks are estimated with and without adjustment for deprivation. Results for methods 2 and 3 are from multiple imputation using 100 datasets (see text for details)

For the first approach, based on the given data, there is a significant increase in risk associated with increased levels of black smoke when using the Poisson model (RR$=$1.037, 95 % CI 1.025 , 1.050). Significant increases in risk are also seen after adjustment for deprivation. Little difference was observed when adjusting for the effects of deprivation. Although this measure of deprivation has been used in many small-area epidemiological studies [15, 17, 18] and has been shown to provide a good measure with which to discriminate between poor health associated with deprivation and vice versa, the score is defined on a national level. To a great extent, the areas studied here, i.e. those that have air pollution monitoring sites located within them, constitute a set of deprived areas. Deprived areas are likely to have higher levels of pollution [19] and thus, due to the practice of locating monitors in locations where pollution might be expected to be highest, these areas are also more likely to be the ones in which monitoring sites are located. In fact, over 70 % of the areas in this study lie in the two most deprived quintiles (over the UK), which would greatly reduce the discriminatory power, in that there would be little to differentiate between a large number of the wards as they would be assigned similar (high) scores.

**Fig. 3:**
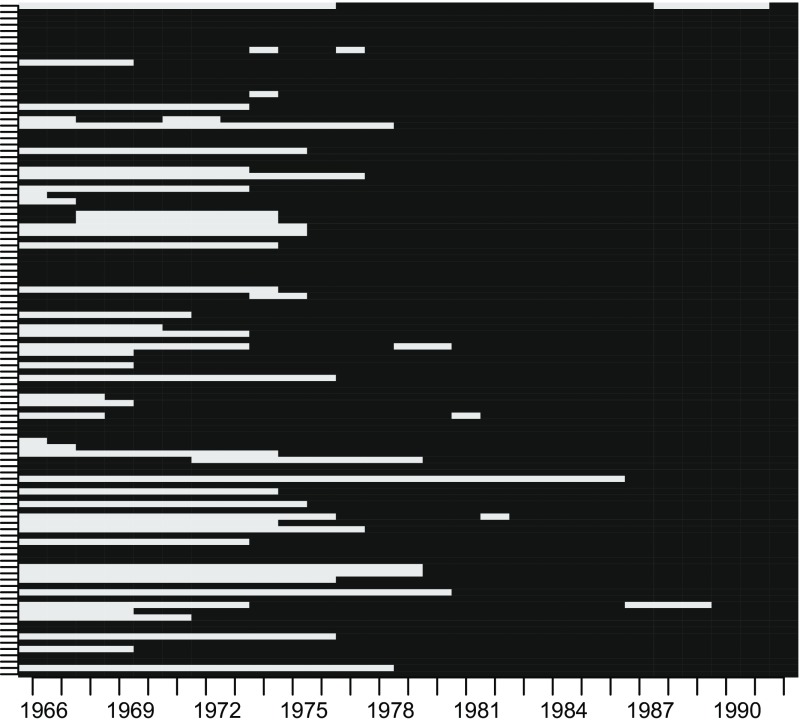
Schematic showing the years for which monitoring sites were operational and those when they were not during the period of exposure; 1966–1992. Data are aggregated to the health area (ward) level. Each line represents a ward with *yellow lines* showing times where there were no operational monitoring sites and *blue lines* where monitoring sites were operational and data available for analysis (Color figure online)

As discussed in Sect. 6.1.2, there is the strong possibility that the results based solely on the available exposure data will be biased if there are strong temporal trends, as in this case when there is a marked decline over time [54]. The availability of the exposure data (at ward level) can be seen in Figure 3, which shows the years for which information was available over the period 1966–1992.

Using approach M2, predictions from the exposure model are used, not just to fill in missing values in the data for times/locations where data were not measured, but also to replace the measurements where they were available. In doing this, as with any model of this type, very high and low values of the exposures will be smoothed towards the mean. However it is precisely the high values that are likely to be driving the health risk and the combination of these high values together with the low ones which will provide the contrast, i.e. the range of values, that is so important for estimation in any regression model. Using this approach, increased risks are observed, RR$=$1.022 (95% CI 1.014–1.030), although the increase is smaller than that observed when using approach M1. The risks again remain after adjustment for deprivation.

To the maximum feasible extent, approach M3 uses a combination of the available data with predictions from the exposure model when measurements are not available. As such, it retains the contrasts in the exposures (unlike approach M2), whilst having a ‘full’ set of data over time for each area, which will reduce the effect of the bias seen in approach M1. In this case, the estimated relative risk is RR$=$1.011 (95 % CI 1.004–1.018).

## Discussion

In this paper, we have incorporated large-scale modelling of air pollution over space and time into epidemiological analyses. In performing epidemiological analyses of the relationship between environmental hazards and adverse health outcomes often there will be locations and periods of time in which exposure information will not be available. This may be due to a fault in monitoring equipment or may be due to the design of monitoring networks and changes over time. In such cases, a direct comparison of the exposure and health outcome is often not possible without an underlying model to align the two in the spatial and temporal domains.

In a fully Bayesian framework, estimation of health and exposure models, including prediction at locations where data are not available, is performed simultaneously. The uncertainty in estimating the coefficients of the exposure model is therefore acknowledged and ‘fed through’ the model to the predictions and further to the estimation of the coefficients in the health model. However, there may be conceptual reasons why ‘feedback’ from the health model to the exposure model is not desired. Here it is the exposures that might be thought of as causing health effects, but the health effects are not thought to affect the exposures in the same way. It is noted that although an epidemiological regression model cannot itself prove causality (that can only really be ascertained by randomised controlled experiments), it can indicate the change in the response variable that might be associated with changes in exposure, either by prediction or estimation, which is a very useful tool in developing insight and understanding into possible causal relationships.

There may also be computational considerations associated with jointly fitting the health and exposure models, especially if the latter uses large amounts of data over space and time. When the exposure model is complicated or when one is interested in running multiple candidate epidemiological models with different sets of covariates, either for a single outcome or multiple outcomes, a single model is not going to provide an efficient method of investigation. A two-stage approach has the advantage that one does not have to refit the exposure model when running multiple health effect analyses. Two-stage approaches separate the exposure and health components, whilst still allowing uncertainty from the exposure modelling to be incorporated into the health model [11, 32, 43]. Here we use multiple imputation based on samples from the joint distribution of the posterior distributions for predictions of the exposures. In approaches M2 and M3, the width of the confidence interval associated with the estimate of risk will incorporate both the uncertainty associated in the estimation of the risk parameter within each of the datasets and also that between the datasets; the latter reflects the uncertainty from the estimation of the exposures.

In the case study, we have attempted to isolate monitoring sites that might indicate the exposures experienced by the populations at risk by selecting only sites that were designated to be in residential areas. However, there is the strong possibility that monitoring sites will have been located in areas that were expected to have high concentrations, as may be the case when assessing whether guidelines and policies are being adhered to. In the context of air pollution and health in epidemiological analyses, Guttorp et al. [27] state that air pollution monitoring sites may be intentionally located for a number of reasons, including to measure: (i) background levels outside of urban areas; (ii) levels in residential areas and (iii) levels near pollutant sources. Shaddick and Zidek [54] show evidence that this was the case for the black smoke network used in the case study. As the number of monitoring sites was reduced over time, those with higher measurements were more likely to be retained. This leads to *preferential sampling* in this example, when the process that determines the locations of the monitoring sites and the process being modelled (concentrations) are in some ways dependent [14]. Zidek et al. [70] showed that there is a significant association between measured levels and the probability of a site remaining in the network. They also presented a method for adjusting summary measures (of the levels of pollution) for changes in the monitoring network and preferential sampling. Future research topics may include the possibility of incorporating adjustments directly into the estimation of health risks.

In theory, it would be relatively straightforward to fit the models considered here using MCMC and this would provide a natural way of allowing the uncertainty associated with using predictions from the exposure model to be fed through to the estimates of the health risks. However, in practice the computational requirements may prove to be prohibitive, both because of the requirement to manipulate large matrixes within each simulation of the MCMC and also in convergence of parameters in complex models. Convergence of the spatial parameters in particular can cause problems [21], especially if datasets are relatively small in which case there might not be enough information to estimate them accurately, although difficulties with accurately estimating spatial parameters with small datasets is of course not exclusive to MCMC.

The models considered here were fit using INLA with the SPDE approach to allow point referenced spatial components to be incorporated. In terms of prediction at a very high number of locations, techniques such as INLA, which perform ‘approximate’ Bayesian inference and thus do not require full MCMC sampling, provide an extremely appealing approach, as shown by Lindgren et al. [36]. In many cases, the underlying field will not be stationary. Bornn et al. showed evidence of non-stationarity in black smoke concentrations [4] and this is likely to occur with air pollution where many factors, such as topography and wind patterns will affect local concentrations. INLA can be extended to cover non-stationary random Gaussian fields and future work will involve integrating predictions from non-stationary exposure models into health models. Overall, the implementation of the INLA and SPDE approaches in this paper demonstrates how the methods can provide a remarkably fast computational algorithm for application over large domains when standard computational methods might fail.
